# The impacts of COVID-19 on China insurance industry—An empirical analysis based on event study

**DOI:** 10.3389/fpubh.2022.1033863

**Published:** 2022-11-28

**Authors:** Xuan Wu, Chan Wang, Hong-xing Wen, Pu-yan Nie, Jin-fa Ye

**Affiliations:** ^1^School of Economics, Guangdong University of Finance and Economics, Guangzhou, China; ^2^Guangzhou Chow Tai Fook Financial Center, Aegon THTF Life Insurance Company, Guangzhou, China

**Keywords:** COVID-19, impacts, insurance industry, event study, abnormal return

## Abstract

**Introduction:**

At the end of 2019, the sudden outbreak of COVID-19 pneumonia has developed from a mass health event to a global epidemic disaster. Its impact extends from human health to social, economic, political, international relations and global governance. In the process of fighting against the epidemic in China, almost all economic sectors were affected, and the insurance industry with epidemic sensitive characteristics was particularly affected.

**Methods:**

In order to identify the impacts of COVID-19 on China's insurance industry, this paper uses the event study method to calculate the changes in the cumulative abnormal return rate and the cumulative excess return of Chinese listed insurance companies before and after the outbreak of COVID-19. In the empirical analysis, five different typical events are examined, including the first outbreak of COVID-19 in China, the closure of Wuhan, the dredging of Wuhan, and the listing of vaccines in China.

**Results:**

The results show that the return rate of listed companies in the insurance industry showed an “inverted N” curve with the “decreasing, rising and then decreasing.” The epidemic mainly has negative effects on the insurance industry in terms of premium income and indemnity expenditure. According to the supply shock theory of the new supply economics, the epidemic has a negative impact on the insurance industry in the short term and a positive impact in the long term.

**Discussion:**

In this context, insurance enterprises should attach importance to the change of business model, strengthen the development model of public-private joint venture insurance, promote product innovation and the application of insurance technology, and the experience and practice of the insurance industry in responding to the impact of the epidemic are of great significance to the transformation of China's insurance industry.

## Introduction

**Figure 1:**
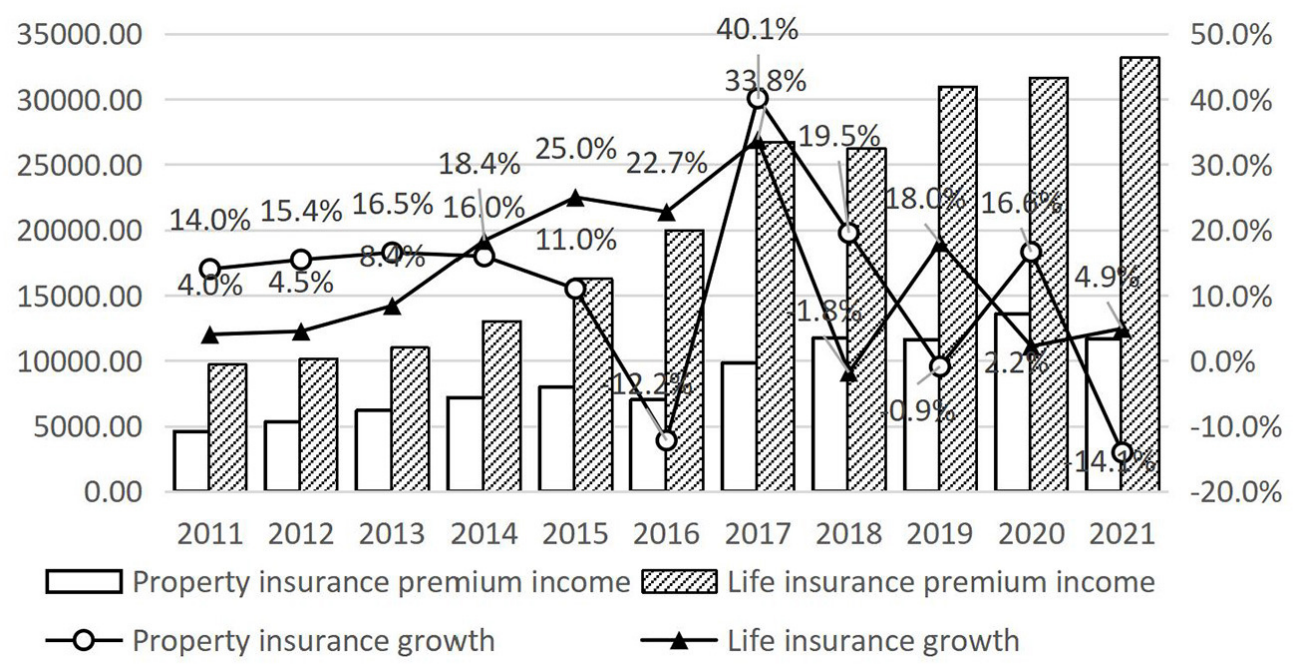
Premium income and growth rate of Property and Life insurance in China from 2011 to 2021. Data source is the Insurance Industry Association of China.

The outbreak of COVID-19 at the end of 2019 has brought a great external impact on the global economy, among which the insurance industry is one of the most sensitive industries hit by the epidemic. According to Allianz Global Insurance Industry Development Report 2021, the global premium income in 2020 was 3.73 trillion euros, 2.1% lower than that in 2019, or about 80 billion euros. In China (see Figure 1), according to the statistics released by the China Insurance Association, the growth rate of life insurance premium income decreased from 18% in 2019 to 2.2% in 2020, while the property insurance premium income in 2021 decreased by 14.1% compared with 2020. If the epidemic continues to break out and is not effectively suppressed, the business development of life insurance based on offline agents and property insurance based on shipping operations will be greatly affected. Compared with developed countries, the epidemic has made developing countries with high foreign trade dependence more impacted on global markets. Due to the shortage of enterprise operating rate in China, a series of insurance types, including property insurance, engineering insurance, employer liability insurance have been affected, and the decline of import and export has directly affected freight insurance. Therefore, accurately quantifying the impact of COVID-19 on the insurance industry is of great important for policymakers to formulate effective.

Due to the sudden outbreak of COVID-19, there are problems with the relevance and completeness of research data. In addition, regression models and scoring systems are mostly used to evaluate the impact of the insurance industry in developed countries. Most of the existing studies only focus on descriptive statistical indicators of economic data during the epidemic and structural impact analysis of changes in the insurance industry, and do not use relevant measurement methods.

The objective of this paper is to investigate the impacts of COVID-19 on China's insurance industry by using the event study method. To this end, we selected six Chinese A-share listed companies as the research samples, from the life insurance industry and the property insurance industry, respectively. These six companies are all representatives of China's insurance industry, and the stock data and financial data they provided are continuous during the research period, which enables us to use the event research method to identify the impacts of COVID-19.

The innovation and contribution of this article are mainly reflected in the following aspects. First, taking China, the second largest insurance market in the world as the research object, to explore the impact of the epidemic on the insurance industry in developing countries; second, using the event research method to specifically analyze the impact of the epidemic on China's insurance industry, and provide important policy guidance and effective suggestions for China to scientifically respond to major emergencies in the future.

The structure of the rest of this article is arranged as follows. Section Literature review provides a literature review. Section Theoretical analysis and research hypothesis presents the impact mechanism of the new crown epidemic on the insurance industry from four aspects: policy environment, consumer psychology, industrial structure adjustment and digital transformation. Section Methods and design introduces the methods and data sources. Section Empirical results and analysis shows the empirical results, followed by the conclusions and policy implications in Section Research implications.

## Literature review

The COVID-19 is a major emergency that has spread most rapidly, has the largest scope, is the most difficult to prevent and control, and has caused the most serious damage to economic development in modern China. Related research is mainly divided into four categories:

First, quantify the time changes of endogenous behavioral responses of economic activities to epidemic from various angles, such as energy consumption social economy, and household consumption. For example, (1) built a basic epidemic model to explain the impact of social isolation and containment policies on the evolution of infectious diseases and their interaction with the economy. Haroon and Rizvi (2) found that the panic caused by the news media after the COVID-19 intensified the turbulence of the global financial market. Wang et al. (3) finds the short term adverse impact of COVID-19 on China's insurance market. Lagoarde-Segot and Leoni (4) found that when malaria and AIDS were both prevalent, the possibility of banking collapse was increasing. Veronica (5) believes that the economic shock related to the company closure and layoffs caused by the COVID-19 has the characteristics of Keynesian supply shock, the change in total demand caused by supply shock is greater than the shock itself. Wu et al. (6) believed that the epidemic situation has increased exponentially in many major cities in China over time.

Second, analyze the impact of blockchain and insurance technology from the perspective of the urgent need for digital transformation in the insurance industry under the epidemic. Eckert et al. (7) found that the digital transformation of insurance is not ahead of the high percentage of intermediary salespeople. Wu (8) found that insurance technology is redefining the insurance industry. ZareRavasan et al. (9) concluded that blockchain technology has the potential to contribute to the digital transformation of business models in various aspects. Grima et al. (10) adopted the STEEP framework to analyze factors that may affect the diffusion and penetration of blockchain in the insurance industry in order to enhance the efficiency and transparency of transactions and settlements. Heini et al. (11) proposed that the most critical digital business enablers are business process automation, online services and big data. Wang (12) believed that insurance Technology had a significant impact on the liability side, asset side and risk-taking behavior.

Third, study the loss distribution of the insurance industry and the application of policy tools under the influence of the epidemic. Gründl et al. (13) analyzed inter temporal risk-sharing scenarios using high-frequency data tracking the economic impact of the COVID-19 pandemic in the United States, and argued that the expected gap in the distribution of epidemic losses could be reduced by 50%. Babuna et al. (14) concluded that the trend is an economic recession with reduced profits and increased claims. Wu et al. (15) believed the severity of the epidemic, the administrative level of cities, and the capabilities of health departments, there are large differences in the statistical data and information of governments at all levels. Liedtke (16) proposes that companies and regulators need to find a balance between policy holder protection and market efficiency good balance.

And last, analyze the transformation of COVID-19 on the business model of the insurance industry. Stojkoski et al. (17) believed that the share of motor vehicle classes in developing countries, has a significant impact on weakening the overall negative impact of the insurance industry. Li et al. (18) used multiple linear regression as variables to construct incremental index, and proposed that national savings and macroeconomic climate index are the main factors affecting the development of China's insurance industry. Li et al. (19) believed that Chinese property insurance companies should take high loss rate and low reinsurance rate as the main development path. Zeyun et al. (20) proposed that the flow of capital, technology and labor between regions may be conducive to the balanced development of insurance between regions.

In summary, the existing literature has analyzed the changes of the insurance industry with descriptive statistical indicators and structural shocks of economic data during the pandemic period, and found that insurance companies have suffered a certain degree of trading losses under the pandemic risk. However, these studies are concentrated in developed markets. The extent to which insurance transactions in emerging markets are affected by the COVID-19 shock has not received much attention. In particular, no literature uses the event study method to identify the impacts on China's insurance industry from the outbreak of COVID-19.

## Theoretical analysis and research hypothesis

Following the basic framework of industry analysis, this paper mainly explains why and how the COVID-19 affects the development of the insurance industry from four aspects: policy environment, consumer psychological changes, industrial structure adjustment and digital transformation.

### Theoretical analysis

#### Change in policy environment under the background of anti-epidemic

China has adopted proactive fiscal and monetary policies to support financial institutions in coping with the impacts of COVID-19. The monthly special reloan issuance rate reduced by 250 basis points from the one-year lending market quoted rate (LPR) of last month. The government also organized insurance companies to jointly launch insurance products to ensure orderly resumption of work and production in all walks of life. For example, in March 2020, Beijing issued management measures and implementation plans for the comprehensive insurance for epidemic prevention and control of enterprises that resumed work and production. This special insurance was insured by a leading insurance company and six member insurance companies, with a fixed premium of 100,000 yuan, 50% of which was subsidized by the government, and corresponding compensation limit was set for the insurance liability.

#### Consumer psychological change

Compared with the policy effects, the changes in consumer psychology and behavior mode under the COVID-19 are more subtle and slow. From January to February 2020, the total retail sales of consumer goods in China decreased by 20.5% year on year, the growth rate was 28.7% lower than that in the same period of the previous year. The sales of automobiles decreased by 37% year on year, and the passenger flow of industries and business types such as accommodation, catering and beauty salons decreased significantly. According to the China Tourism Academy, due to the COVID-19, the number of domestic tourist trips in the first quarter and the whole year of 2020 decreased by 56% and 15.5%, respectively, with a year-on-year decrease of 932 million. In terms of property insurance industry, due to the needs of epidemic prevention and control, the transportation, tourism and catering industries are greatly impacted, and the public's awareness of property insurance demand is not growing as fast as that of personal insurance demand.

#### Adjustment of the insurance industry structure

**Figure 2:**
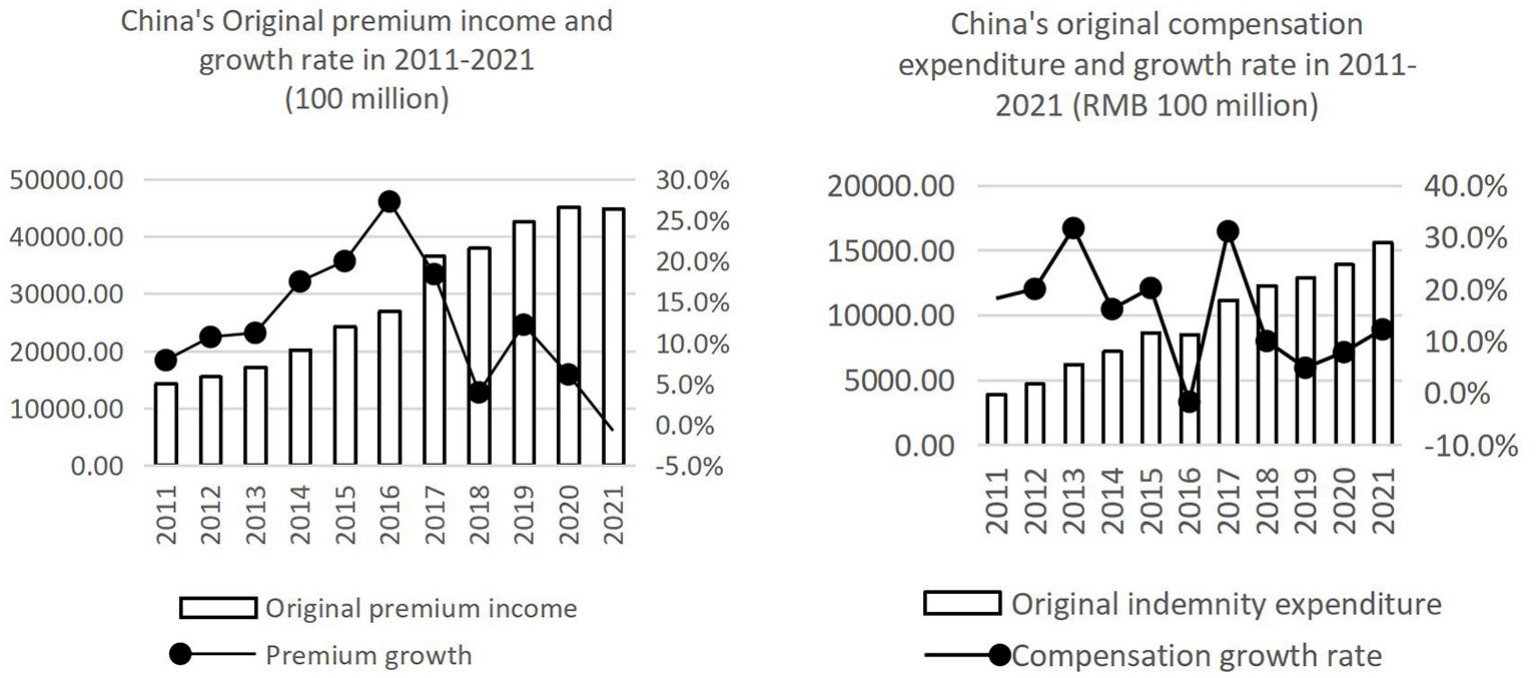
Original premium income and compensation expenditure in China from 2011 to 2021. Data source is Insurance Industry Association of China.

Under the multiple changes of policy environment and consumer psychology and behavior, China's insurance structure has undergone significant adjustment. This paper selected and investigated the data comparison of China's insurance industry before and after the outbreak of COVID-19 (see Figure 2), and conducted further analysis. In 2020, China's original premium income was 4.52 trillion yuan, up 6.11% year on year; Compensation payments amounted to 1.39 trillion yuan, up 7.9 percent year on year and slightly higher than in 2019. After the outbreak of the epidemic, although the outbreak of insurance claims and operating costs have an impact. However, it also provides an opportunity for the restructuring of the insurance industry. Therefore, the epidemic will accelerate the improvement of online channels of insurance enterprises, accelerate the pace of science and technology to help smart insurance and insurance product innovation, and further highlight the attributes of Internet tools to accelerate the online layout.

#### Digital transformation of insurance

The globalization of economy and finance is an irreversible trend of the times. As an important part of finance and a basic means of risk management in the modern economy, the trend of digitizing of insurance cannot be changed. Driven by the strong digital economy, the insurance industry, which regards customer data as its most valuable asset, is rapidly transforming into digitizing. Actively promoting the integration of big data, cloud computing, artificial intelligence and other insurance technologies with the insurance industry, and exploring the effective integration of online and offline will be the key to future industry development trends and companies to win opportunities. Therefore, the integration of modern insurance and technology has become the general trend. Technology empowerment has triggered profound changes in the industry, leading the insurance industry to develop steadily in a highly market-oriented competitive environment.

### Research hypothesis

Combined with the above theoretical analysis, China's economy has encountered the COVID-19 during the transition period of high-quality development, and insurance is an important basis and means for the country's macro leadership, regulation, and optimization of economic development. According to the new supply-side economics, the supply shock, which is defined by the new supply-side economics, mainly acts on the external events on the supply side, such as production factors and supply chain, in addition to the factors affecting the normal economic cycle and growth trend. In view of this, this paper proposes two research hypotheses:

Hypothesis 1: The COVID-19 has had a short-term negative impact on the insurance industry.

On the one hand, due to the epidemic, insurance companies are unable to carry out offline marketing, and premium income has dropped significantly compared with the same period of the previous year. The new business development of insurance companies is mainly through the offline development of insurance agents or marketers. Due to the impact of the epidemic, marketers were unable to carry out offline business expansion. On the other hand, the reduction of new business and the turmoil in the financial market have made it more difficult for insurance companies to allocate funds. Due to the expected sharp drop in premium income at the beginning of the year, the growth rate of available funds for insurance funds has also slowed down significantly. The scale of capital inflow of insurance companies has declined, which is a test for the liquidity of insurance companies.

Hypothesis 2: The COVID-19 has had a long positive impact on the insurance industry.

In the long term, the COVID-19 is an important opportunity for the development of the insurance industry. Insurance gradually turns from a single risk transfer function to participate in the whole process management of risk identification, risk assessment and risk response. After the pandemic, the public will pay more attention to their own health management and insurance awareness will grow. The epidemic has also enabled insurance companies to accumulate experience in operation, management and risk control in actual combat. Insurance companies have developed new types of insurance and accelerated the application of insurance technology to improve the efficiency of insurance business. This opens up new premium growth opportunities in areas such as property, engineering and warranty insurance.

Therefore, although the COVID-19 has brought negative impact in the short term, its specific impact needs to comprehensively consider the impact of alternative growth, innovative growth, compensatory growth and government support and stimulus policies. In the medium term, in addition to shrinking demand, the impact of the epidemic will also accelerate the expansion of new supply and the withdrawal of aging supply, which is conducive to the arrival of the next new supply expansion cycle. In the long run, the impact of COVID-19 will increase and improve the development opportunities of the insurance industry, and the impact on the long-term growth of the insurance industry is positive.

## Methods and design

### Research methods

This article mainly uses the event study method to empirically analyze the impacts of COVID-19 on China's insurance industry. Based on the counterfactual framework, the event research method analyzes the prices of insurance companies in the securities market before and after the occurrence of specific events, and tests whether there are abnormal returns (AR). The basic assumption of the event research method is that the market is valid, and the impact of the event is immediately reflected in the insurance company's income. So observing short-term fluctuations in an insurance company's earnings can measure the economic impact of an event. Since Dos Santos et al. (21) first analyzed the impact of IT investment on stock market yields, this method has been widely used in analyzing the value appreciation or depreciation created by certain events. This point brings confidence to researchers to use the event research method to analyze Chinese problems, which can be seen from the increasing number of such literature results in recent years.

The event study method is mainly applied to the calculation of the normal return rate and the abnormal return rate. The normal return is to assume the “normal” expected return of the company's stock in the absence of the event. It should be clear that the adoption of stock price changes is based on the investor point of view rather than the business perspective (e.g., corporate profits). This index has the following advantages: First, high content validity. Share price changes on the surface is a measure of investors' earnings, but in a relatively sound capital market, investors can “vote with their feet,” make this index can cover the listed company operating profits, risk, asset structure, competitive environment, become a comprehensive index to measure its sustainable development prospects, more suitable for the investigation of the development of the insurance industry. Secondly, good measurement. The series of COVID-19 events analyzed in this paper mainly occurred in 2020. The financial income data statistics cycle of listed companies is generally quarterly or annual, while the stock return rate is daily data, which can capture the immediate impact of specific events in a short period of time.

The core of the event research method is to measure and analyze abnormal returns, that is, the abnormal movements that cause the stock market to deviate from the normal state due to the occurrence of events:


$$\begin{array}{l} AR_{t}=R_{t}-ER_{t} \end{array}$$

Among them, R_t_ is the actual return of the underlying asset, calculated based on actual data; ER_t_ is the normal return, that is, the expected return of the underlying asset when the stock market is operating normally, and the difference between the two is abnormal return AR_t_.

Since the research sample of this article is market index income, the normal income calculation adopts the normal mean income model. The idea of this model is to use the average income of the underlying asset in the estimation window as the normal income in the event window.


$$\begin{array}{l} R_{t}=\mu +\varepsilon_{t} \end{array}$$

μ is the average return of the underlying asset in the estimation window and ε_*t*_ is a random perturbation term with zero mean and equal variance.

After calculating the daily abnormal income *AR*_*t*_ in the event window, the cumulative excess return rate is vertically added up to *CAR*_*t*_, reflect the overall impact of the event on the income of the underlying assets.


$$\begin{array}{l} {CAR}_{t}=\overset{t}{\underset{-1}{\sum }}{AR}_{t},\;t\in \left[-1,1\right] \end{array}$$

After the abnormal gain was obtained, the significance was tested by the T statistic. Null hypothesis, *H*_0_ : *AR*_*t*_ = 1, alternative hypothesis. *H*_1_ : *AR*_*t*_ = 0.

### Sample selection and data source

In terms of research samples, this article selects six Chinese A-share listed companies in China's insurance industry as the research objects, including China Pacific Insurance, China Ping An, China Life, PICC, New China Insurance and Tianmao Group, of which Tianmao Group, New China Insurance, China Life is mainly engaged in life insurance business, China Pacific Insurance and China Ping An are comprehensive insurance companies, and PICC is mainly in property insurance. The above companies all have complete stock data and financial data during the study period. The market model estimates the normal rate of return by using the comprehensive rate of return of the securities market. In this paper, the Shanghai Composite Index is used as the research sample of the event study method to reflect the impact of the outbreak on the insurance market as a whole. The data used are from the CSMAR database.

Event selection. This article follows the principles of relevance, suddenness, and independence, and selects events related to the new crown epidemic—the first confirmed case of new coronary pneumonia in China on November 7, 2019, the closure of Wuhan on January 23, 2020, and April 8, 2020. The unblocking of Wuhan and the launch of China's new coronavirus vaccine on December 31, 2020 were studied for four events. In order to describe the characteristics of the events, the variable Time was constructed based on the time interval between the day of the event and the first event, and the unit is day. This article uses all stock data and basic information of listed companies from CSMAR database, Google Finance database, Yahoo Finance database and Oriental Fortune.com. The event information comes from media reports such as Caixin's Global New Crown Anti-epidemic Events, Sina.com's New Crown Campaign Review (epidemic events), and New Coronary Pneumonia Event Timeline by Events.com, as well as related documents on CNKI.

## Empirical results and analysis

### Data stability test

This article uses the ADF statistic to perform unit root test on the returns of 6 sample listed companies on all trading days from July 1, 2019 to June 1, 2021, and selects the term with intercept, without time trend and lag. The results show that the time series of all returns reject the null hypothesis of the existence of a unit root at the 1% significance level (see Supplementary material for the test results), indicating that the time series is stationary and can be used for empirical analysis.

### Stock price trend

**Figure 3:**
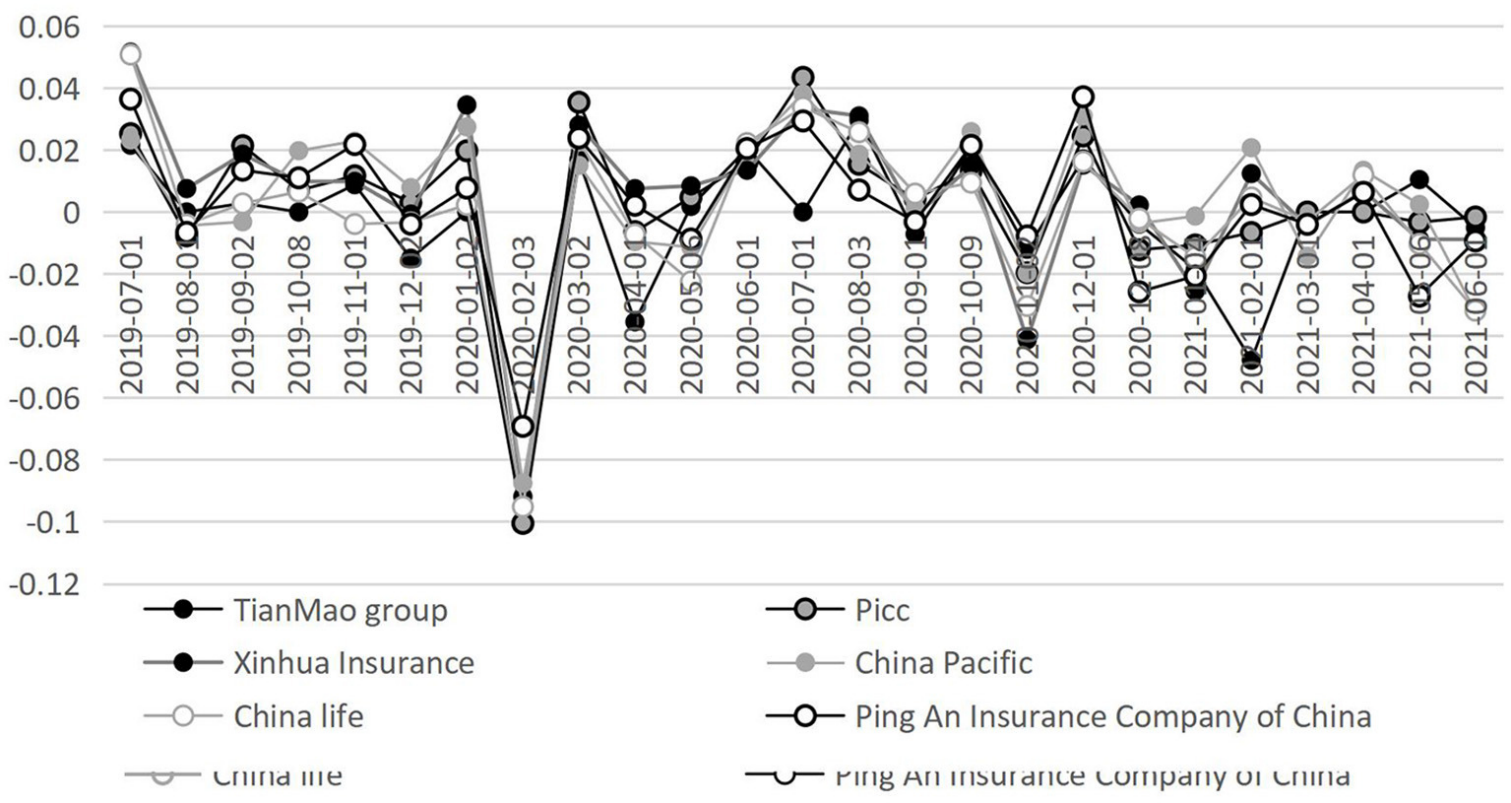
Analysis of the circulating market value of listed companies in China's insurance industry. Data source: CSMAR database.

The changes in the insurance total circulation market value of listed companies before and after the outbreak of COVID-19 are shown in Figure 3. It can be clearly seen that the change trend of circulation value of life insurance, insurance and comprehensive insurance is similar. The circulation market value began to decline gradually at the end of 2019, reached the bottom in April 2020, then slowly fell back and reached the peak in October 2020, and then quickly fell back again.

**Figure 4:**
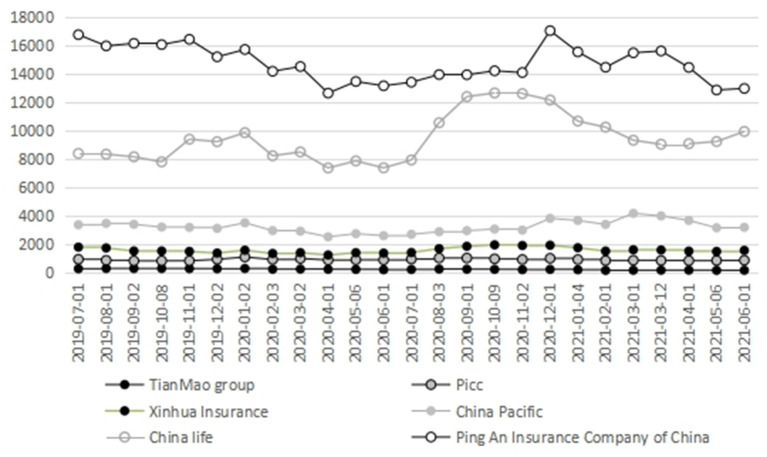
Analysis of the returns of listed companies in China's insurance industry. Data source: CSMAR database.

As shown in Figure 4, the yield expressed an “inverted N” fluctuation trend after the outbreak of the COVID-19. Specifically, it declined rapidly after reaching its peak on January 8, 2020, reached its trough in March, and then rose rapidly and remained at a high level. The sharp decline in the two time series is basically consistent with the outbreak time of the domestic epidemic in China. When the domestic epidemic situation is basically controlled, and work, production, and school are resumed one after another, the domestic consumer demand suppressed in the early stage is partially released, and the insurance industry ushered in a strong momentum of development.

### Effect of different events on AR_t_

Before measuring the impact of an event on stock prices, we need to determine the event window and the estimation window. First, determine the date of the first confirmed COVID-19 incident in China. If the stock market is a trading day, it will be regarded as the 10th day. If it is a non-trading day, the first trading day will be the 10th day. In this article, the event window was set for 30 trading days, from the 15th trading day to the 15th trading day after the 0th trading day, which is expressed as [−15, 15]. The estimation window was set for 499 trading days, from the 120th trading day to the 379th trading day after the 0th trading day, which is expressed as [−120, 379].

This paper conducts a cross-check of events on 6 insurance stocks in China. Statistical analysis display that before the outbreak of the new crown epidemic, the stock market returns were in normal fluctuations, and the number of trading days with positive and negative abnormal returns was basically the same. The abnormal returns of the Shanghai Composite Index began to be significantly negative on the sixth trading day after the outbreak, the decline further expanded on the eighth and ninth trading days, and began to bottom out after the tenth trading day, but it continued In the small fluctuation, there is no major sign of recovery, which shows that the impact of the outbreak on the stock market has a certain lag. From the analysis of abnormal returns alone, the stock market has clearly responded to the stimulus of Wuhan's lockdown, with irregular fluctuations before and after the event; the stimulus of Wuhan's unblocking coincided with the global spread of the epidemic, and the global stock market was in a mess, subject to the pessimistic expectations of the global economy, the stock market There has not been a very significant surge in the response to China's new crown vaccine; the market has been stimulated by the launch of China's new crown vaccine. Before this event, the stock market fell significantly for 4 consecutive trading days, but it was also the one with the strongest response. After the event, it began to rise continuously, and in the 15 trading days There were 13 trading days where abnormal returns were positive and 9 were significantly positive, reflecting a strong positive trend. As shown in Table 1, the average cumulative abnormal return CAR of all stocks in [−15, 15] for a total of 30 trading days is −0.0096<0, and it is significant at the 1% level, indicating that the new crown epidemic event during the research period has brought a negative impact on Chinese insurance stocks as a whole to abnormal returns.

**Table 1:** Abnormal returns: AR.

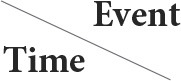	**The outbreak**	**Wuhan closed**	**Wuhan unsealed**	**Vaccine launched**
15	0.003723	−0.004736	0.004952**	0.013795***
14	0.004908*	0.001182	0.006577***	0.017309***
13	0.003152	0.016441***	0.003118*	0.00368
12	0.017037***	−0.005166	−0.0057	0.057056***
11	0.012359***	−0.001507	0.015891***	0.02008***
10	0.01321***	0.020875***	−0.004857	0.021287***
9	−0.077389***	0.001805	−0.010359***	0.013783***
8	−0.027661***	−0.009077**	0.003739**	0.007759
7	0.002676	0.006735***	−0.001912	−0.006109***
6	−0.014244***	0.001908	0.020538***	0.002948
5	0.006453**	0.003092*	−0.005989	0.001746**
4	0.000317	0.001337	0.016864***	−0.000853***
3	−0.005309	0.015222***	−0.005739	0.009573
2	−0.005546	0.010543***	0.001123	0.001117**
1	−0.002953	0.011395***	−0.009016**	0.001347**
0	0.007384***	−0.079204***	0.002635*	0.014378***
-1	−0.000981	−0.029476***	−0.005998	−0.010234***
-2	0.008982***	0.000861	0.021725***	−0.000455***
-3	−0.012355***	−0.01606***	0.023407***	−0.00782***
-4	0.006793**	0.004637**	−0.031124***	−0.004239***
-5	−0.000268	−0.001498	0.016093***	0.006185
-6	−0.000602	−0.007125	−0.009761**	0.00232*
-7	0.011357***	−0.007361	−0.018306***	0.003898
-8	0.003178	−0.004768	−0.003447	−0.001468***
-9	0.011496***	0.005569**	−0.034004***	0.000616**
-10	−0.000913	−0.002797	−0.012336***	0.00199*
-11	0.008397***	0.007167***	−0.015171***	0.022057***
-12	−0.000413	−0.01417***	−0.009425**	0.002096*
-13	0.006582**	0.004977**	0.018165***	0.003262
-14	−0.014185***	−0.002083	−0.030062***	−0.003483***

* **, ** and * represent those passing the significance level test of 1, 5, and 10%, respectively.

### Effect of different events on CAR_t_

**Figure 5:**
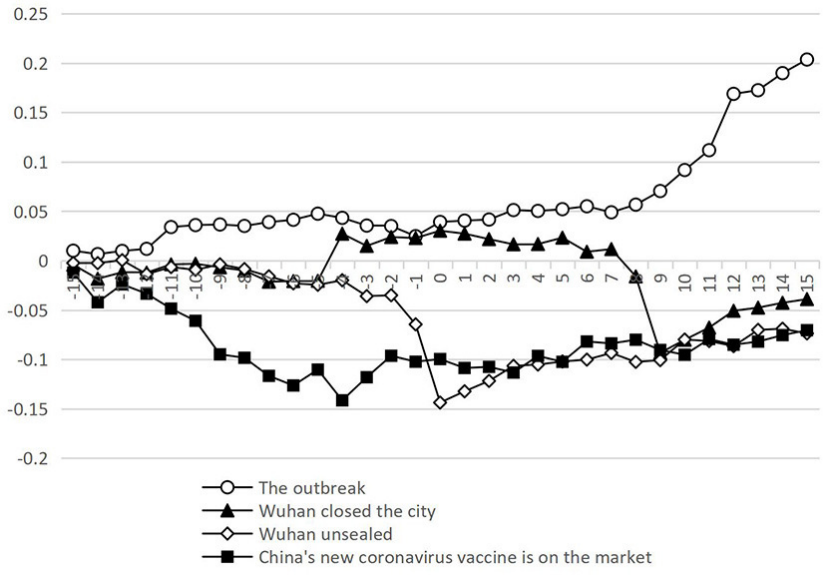
Accumulated excess yield rate. CAR_t_.

Figure 5 presents the changes of cumulative excess yield obtained from the vertical aggregation of abnormal returns. As can be seen, the stock market showed an upward trend before the outbreak of the epidemic, and began to decline gradually after the outbreak, but it was still in a small range of fluctuations. This indicates that the impacts of the epidemic on the stock market had a short time lag effect. However, with the increases of confirmed cases, the impacts of the epidemic on economic activities began to show clearly. The stock market began to fall sharply on the fourth day after the outbreak of the epidemic until it researched the bottom.

## Research implications

In this article, the event research method was used to investigate the impacts of the COVID-19 on China's insurance industry. The empirical evidence shows the epidemic mainly has negative effects on the insurance industry in terms of premium income and indemnity expenditure, resulting the return rate of listed companies in the insurance industry showed an “inverted N” curve. Although our findings are negative, it should be noted that the policy environment, industrial organization structure and business model of China's insurance industry are undergoing tremendous changes after the outbreak of COVID-19. We believe that China's insurance industry will benefit from these changes in the future. Therefore, from a long-term perspective, the impacts of the COVID-19 epidemic on China's insurance industry may be both threats and opportunities.

According to our findings, the following policy recommendations may be beneficial to promoting the healthy development of China's insurance industry during the post-epidemic period.

First, integrate industry forces and strengthen multi agency cooperation. After the impact of the epidemic, the public's awareness of health insurance will be further strengthened. However, due to the high professional requirements in the medical field, insurance companies should strengthen cooperation with medical and health research institutions to carry out research on risk identification and management of new infectious diseases. Combining the government, hospitals, pharmaceutical enterprises and insurance companies through the Internet platform to build a healthy community.

Second, optimize the financing structure and improve solvency management. Under the background of the COVID-19, the average comprehensive solvency adequacy disclosed by all major direct insurance companies and reinsurance companies exceeded 200%, significantly higher than the minimum regulatory requirement of 100%. However, due to the deterioration of the investment environment, insurance companies should further optimize their financing structure.

Third, improve risk management and strengthen crisis management. During the impact of the COVID-19, the income and compensation expenditure of insurance companies have fluctuated greatly. Insurance companies need to strengthen crisis management, pay attention to changes in interest rates, decreases in asset values, increases in claims and other adverse events. In addition, in order to cope with the impact of public health emergencies, a crisis management system needs to be established to assess the impact of the epidemic on capital levels, investment portfolios and other risk factors.

And last, use insurance technology to realize digital transformation. During the epidemic, China's insurance industry exposed the problem of relying heavily on offline models. Insurance companies should achieve digital transformation, establish digital channels to build customer service platforms, optimize the customer relationship management system, improve the current situation of missing contact information and duplicate entries. Technical innovation should aim at market demand and design insurance products or services that are more suitable for new channels or new marketing models.

## Data availability statement

The datasets presented in this study can be found in online repositories. The names of the repository/repositories and accession number(s) can be found below: http://www.iachina.cn/col/col41/index.html insurance association of China https://cn.gtadata.com/ Stock Market and Accounting Research Database.

## Author contributions

XW: main methods of the article and the writing of the literature review. CW: method refinement. H-xW: text refinement. P-yN: idea guidance. J-fY: data search. All authors contributed to the article and approved the submitted version.

## Funding

This work is partially supported by the National Natural Science Foundation of PRC (72003044 and 72003045), Natural Science Foundation of Guangdong Province (2022B1515020034, 2022A1515011903, and 2021A1515011960), and Guangzhou Social Science Project (2020GZQN39).

## Conflict of interest

Author J-fY was employed by the company Aegon THTF Life Insurance Company. The remaining authors declare that the research was conducted in the absence of any commercial or financial relationships that could be construed as a potential conflict of interest.

## Publisher's note

All claims expressed in this article are solely those of the authors and do not necessarily represent those of their affiliated organizations, or those of the publisher, the editors and the reviewers. Any product that may be evaluated in this article, or claim that may be made by its manufacturer, is not guaranteed or endorsed by the publisher.
