# Supplementary material for: The impacts of COVID-19 on China insurance industry—An empirical analysis based on event study
Source: Front Public Health. 2022 Nov 28;10:1033863. doi: 10.3389/fpubh.2022.1033863 (PMC9742270; doi:10.3389/fpubh.2022.1033863)
Supplement: Supplementary file 1 [file Table_1.DOCX]

Supplementary Material

**APPENDIX**

Attached Table 1. The Stationarity Test of Tianmao Group's Trading Day Returns Without Constants and Trends

| Null Hypothesis: ________01 has a unit root | | | |  |
| --- | --- | --- | --- | --- |
|  |  |  |  |  |
|  |  |  |  |  |
|  |  |  | t-Statistic | Prob.* |
|  |  |  |  |  |
|  |  |  |  |  |
| Augmented Dickey-Fuller test statistic | | | -24.06071 | 0.0000 |
| Test critical values: | 1% level |  | -2.570192 |  |
|  | 5% level |  | -1.941540 |  |
|  | 10% level |  | -1.616219 |  |
|  |  |  |  |  |
|  |  |  |  |  |
| Dependent Variable: D(________01) | | | |  |
|  |  |  |  |  |
|  |  |  |  |  |
| Variable | Coefficient | Std. Error | t-Statistic | Prob. |
|  |  |  |  |  |
|  |  |  |  |  |
| ________01(-1) | -1.080627 | 0.044913 | -24.06071 | 0.0000 |
|  |  |  |  |  |
|  |  |  |  |  |
| R-squared | 0.567049 | Mean dependent var | | -0.000116 |
| Adjusted R-squared | 0.567049 | S.D. dependent var | | 0.028777 |
| S.E. of regression | 0.018935 | Akaike info criterion | | -5.093326 |
| Sum squared resid | 0.158477 | Schwarz criterion | | -5.084085 |
| Log likelihood | 1129.172 | Hannan-Quinn criter. | | -5.089682 |
| Durbin-Watson stat | 1.935967 |  |  |  |
|  |  |  |  |  |
|  |  |  |  |  |

Attached Table 2. The first-order difference test of Tianmao Group's daily return without constant and trend terms

| Null Hypothesis: D(________01) has a unit root | | | | |
| --- | --- | --- | --- | --- |
|  |  |  |  |  |
|  |  |  |  |  |
|  |  |  | t-Statistic | Prob.* |
|  |  |  |  |  |
|  |  |  |  |  |
| Augmented Dickey-Fuller test statistic | | | -11.37222 | 0.0000 |
| Test critical values: | 1% level |  | -2.572277 |  |
|  | 5% level |  | -1.941827 |  |
|  | 10% level |  | -1.616030 |  |
|  |  |  |  |  |
|  |  |  |  |  |
| Dependent Variable: D(________01,2) | | | |  |
|  |  |  |  |  |
|  |  |  |  |  |
| Variable | Coefficient | Std. Error | t-Statistic | Prob. |
|  |  |  |  |  |
|  |  |  |  |  |
| D(________01(-1)) | -5.761348 | 0.506616 | -11.37222 | 0.0000 |
| D(________01(-1),2) | 3.821028 | 0.479104 | 7.975369 | 0.0000 |
| D(________01(-2),2) | 2.989637 | 0.430438 | 6.945564 | 0.0000 |
| D(________01(-3),2) | 2.264887 | 0.370077 | 6.120041 | 0.0000 |
| D(________01(-4),2) | 1.638554 | 0.304954 | 5.373119 | 0.0000 |
| D(________01(-5),2) | 1.149739 | 0.237543 | 4.840129 | 0.0000 |
| D(________01(-6),2) | 0.702609 | 0.170790 | 4.113884 | 0.0000 |
| D(________01(-7),2) | 0.363283 | 0.106401 | 3.414268 | 0.0007 |
| D(________01(-8),2) | 0.168854 | 0.047307 | 3.569360 | 0.0004 |
|  |  |  |  |  |
|  |  |  |  |  |
| R-squared | 0.840297 | Mean dependent var | | -4.02E-05 |
| Adjusted R-squared | 0.836148 | S.D. dependent var | | 0.046888 |
| S.E. of regression | 0.018980 | Akaike info criterion | | -5.062904 |
| Sum squared resid | 0.110952 | Schwarz criterion | | -4.956184 |
| Log likelihood | 811.4702 | Hannan-Quinn criter. | | -5.020275 |
| Durbin-Watson stat | 2.046228 |  |  |  |
|  |  |  |  |  |
|  |  |  |  |  |

Attached Table 3.The Stationarity Test of PICC’s Trading Day Returns Without Constants and Trends

| Null Hypothesis: ________02 has a unit root | | | |  |
| --- | --- | --- | --- | --- |
|  |  |  |  |  |
|  |  |  |  |  |
|  |  |  | t-Statistic | Prob.* |
|  |  |  |  |  |
|  |  |  |  |  |
| Augmented Dickey-Fuller test statistic | | | -13.24058 | 0.0000 |
| Test critical values: | 1% level |  | -2.570216 |  |
|  | 5% level |  | -1.941543 |  |
|  | 10% level |  | -1.616217 |  |
|  |  |  |  |  |
|  |  |  |  |  |
| Dependent Variable: D(________02) | | | |  |
|  |  |  |  |  |
|  |  |  |  |  |
| Variable | Coefficient | Std. Error | t-Statistic | Prob. |
|  |  |  |  |  |
|  |  |  |  |  |
| ________02(-1) | -0.883834 | 0.066752 | -13.24058 | 0.0000 |
| D(________02(-1)) | -0.120361 | 0.045842 | -2.625550 | 0.0090 |
|  |  |  |  |  |
|  |  |  |  |  |
| R-squared | 0.510260 | Mean dependent var | | -7.93E-06 |
| Adjusted R-squared | 0.509145 | S.D. dependent var | | 0.025873 |
| S.E. of regression | 0.018127 | Akaike info criterion | | -5.178277 |
| Sum squared resid | 0.144254 | Schwarz criterion | | -5.159732 |
| Log likelihood | 1143.810 | Hannan-Quinn criter. | | -5.170962 |
| Durbin-Watson stat | 2.033342 |  |  |  |
|  |  |  |  |  |
|  |  |  |  |  |

Attached Table 4. The first-order difference test of PICC’s daily returns without constant and trend terms

| Null Hypothesis: D(________02) has a unit root | | | | |
| --- | --- | --- | --- | --- |
|  |  |  |  |  |
|  |  |  |  |  |
|  |  |  | t-Statistic | Prob.* |
|  |  |  |  |  |
|  |  |  |  |  |
| Augmented Dickey-Fuller test statistic | | | -16.24973 | 0.0000 |
| Test critical values: | 1% level |  | -2.570727 |  |
|  | 5% level |  | -1.941613 |  |
|  | 10% level |  | -1.616171 |  |
|  |  |  |  |  |
|  |  |  |  |  |
| Dependent Variable: D(________02,2) | | | |  |
|  |  |  |  |  |
|  |  |  |  |  |
| Variable | Coefficient | Std. Error | t-Statistic | Prob. |
|  |  |  |  |  |
|  |  |  |  |  |
| D(________02(-1)) | -2.834398 | 0.174427 | -16.24973 | 0.0000 |
| D(________02(-1),2) | 0.957969 | 0.144486 | 6.630170 | 0.0000 |
| D(________02(-2),2) | 0.419285 | 0.097012 | 4.321991 | 0.0000 |
| D(________02(-3),2) | 0.152592 | 0.045810 | 3.330967 | 0.0009 |
|  |  |  |  |  |
|  |  |  |  |  |
| R-squared | 0.829352 | Mean dependent var | | 0.000263 |
| Adjusted R-squared | 0.828066 | S.D. dependent var | | 0.046925 |
| S.E. of regression | 0.019457 | Akaike info criterion | | -5.031284 |
| Sum squared resid | 0.150678 | Schwarz criterion | | -4.991518 |
| Log likelihood | 1015.288 | Hannan-Quinn criter. | | -5.015539 |
| Durbin-Watson stat | 2.010103 |  |  |  |
|  |  |  |  |  |
|  |  |  |  |  |

Attached Table 5. Stationarity Test of Xinhua Insurance’s Trading Day Returns Without Constants and Trends

| Null Hypothesis: ________03 has a unit root | | | |  |
| --- | --- | --- | --- | --- |
|  |  |  |  |  |
|  |  |  |  |  |
|  |  |  | t-Statistic | Prob.* |
|  |  |  |  |  |
|  |  |  |  |  |
| Augmented Dickey-Fuller test statistic | | | -23.01496 | 0.0000 |
| Test critical values: | 1% level |  | -2.570066 |  |
|  | 5% level |  | -1.941522 |  |
|  | 10% level |  | -1.616231 |  |
|  |  |  |  |  |
|  |  |  |  |  |
| Dependent Variable: D(________03) | | | |  |
|  |  |  |  |  |
|  |  |  |  |  |
| Variable | Coefficient | Std. Error | t-Statistic | Prob. |
|  |  |  |  |  |
|  |  |  |  |  |
| ________03(-1) | -1.050062 | 0.045625 | -23.01496 | 0.0000 |
|  |  |  |  |  |
|  |  |  |  |  |
| R-squared | 0.539018 | Mean dependent var | | -7.02E-05 |
| Adjusted R-squared | 0.539018 | S.D. dependent var | | 0.033303 |
| S.E. of regression | 0.022612 | Akaike info criterion | | -4.738509 |
| Sum squared resid | 0.231611 | Schwarz criterion | | -4.729438 |
| Log likelihood | 1076.642 | Hannan-Quinn criter. | | -4.734935 |
| Durbin-Watson stat | 1.982340 |  |  |  |
|  |  |  |  |  |
|  |  |  |  |  |

Attached table 6. First-order difference test of Xinhua Insurance’s daily return without constant and trend terms

| Null Hypothesis: D(________03) has a unit root | | | | |
| --- | --- | --- | --- | --- |
|  |  |  |  |  |
|  |  |  |  |  |
|  |  |  | t-Statistic | Prob.* |
|  |  |  |  |  |
|  |  |  |  |  |
| Augmented Dickey-Fuller test statistic | | | -18.26417 | 0.0000 |
| Test critical values: | 1% level |  | -2.570727 |  |
|  | 5% level |  | -1.941613 |  |
|  | 10% level |  | -1.616171 |  |
|  |  |  |  |  |
|  |  |  |  |  |
| Dependent Variable: D(________03,2) | | | |  |
|  |  |  |  |  |
|  |  |  |  |  |
| Variable | Coefficient | Std. Error | t-Statistic | Prob. |
|  |  |  |  |  |
|  |  |  |  |  |
| D(________03(-1)) | -3.145245 | 0.172209 | -18.26417 | 0.0000 |
| D(________03(-1),2) | 1.287670 | 0.141747 | 9.084306 | 0.0000 |
| D(________03(-2),2) | 0.660789 | 0.096097 | 6.876248 | 0.0000 |
| D(________03(-3),2) | 0.231800 | 0.046461 | 4.989086 | 0.0000 |
|  |  |  |  |  |
|  |  |  |  |  |
| R-squared | 0.817742 | Mean dependent var | | 2.42E-06 |
| Adjusted R-squared | 0.816368 | S.D. dependent var | | 0.058456 |
| S.E. of regression | 0.025050 | Akaike info criterion | | -4.526014 |
| Sum squared resid | 0.249739 | Schwarz criterion | | -4.486248 |
| Log likelihood | 913.7288 | Hannan-Quinn criter. | | -4.510269 |
| Durbin-Watson stat | 2.069479 |  |  |  |
|  |  |  |  |  |
|  |  |  |  |  |

Attached Table 7. Stationarity Test of CPIC Trading Daily Returns Without Constants and Trends

| Null Hypothesis: ________04 has a unit root | | | |  |
| --- | --- | --- | --- | --- |
|  |  |  |  |  |
|  |  |  |  |  |
|  |  |  | t-Statistic | Prob.* |
|  |  |  |  |  |
|  |  |  |  |  |
| Augmented Dickey-Fuller test statistic | | | -22.22606 | 0.0000 |
| Test critical values: | 1% level |  | -2.570066 |  |
|  | 5% level |  | -1.941522 |  |
|  | 10% level |  | -1.616231 |  |
|  |  |  |  |  |
|  |  |  |  |  |
| Dependent Variable: D(________04) | | | |  |
|  |  |  |  |  |
|  |  |  |  |  |
| Variable | Coefficient | Std. Error | t-Statistic | Prob. |
|  |  |  |  |  |
|  |  |  |  |  |
| ________04(-1) | -1.028957 | 0.046295 | -22.22606 | 0.0000 |
|  |  |  |  |  |
|  |  |  |  |  |
| R-squared | 0.521637 | Mean dependent var | | -0.000133 |
| Adjusted R-squared | 0.521637 | S.D. dependent var | | 0.031091 |
| S.E. of regression | 0.021504 | Akaike info criterion | | -4.838972 |
| Sum squared resid | 0.209474 | Schwarz criterion | | -4.829901 |
| Log likelihood | 1099.447 | Hannan-Quinn criter. | | -4.835398 |
| Durbin-Watson stat | 1.994641 |  |  |  |
|  |  |  |  |  |
|  |  |  |  |  |

Attached Table 8. The first-order difference test of CPIC’s daily returns without constant and trend terms

| Null Hypothesis: D(________04) has a unit root | | | | |
| --- | --- | --- | --- | --- |
|  |  |  |  |  |
|  |  |  |  |  |
|  |  |  | t-Statistic | Prob.* |
|  |  |  |  |  |
|  |  |  |  |  |
| Augmented Dickey-Fuller test statistic | | | -16.84561 | 0.0000 |
| Test critical values: | 1% level |  | -2.570727 |  |
|  | 5% level |  | -1.941613 |  |
|  | 10% level |  | -1.616171 |  |
|  |  |  |  |  |
|  |  |  |  |  |
| Dependent Variable: D(________04,2) | | | |  |
|  |  |  |  |  |
|  |  |  |  |  |
| Variable | Coefficient | Std. Error | t-Statistic | Prob. |
|  |  |  |  |  |
|  |  |  |  |  |
| D(________04(-1)) | -2.940687 | 0.174567 | -16.84561 | 0.0000 |
| D(________04(-1),2) | 1.113067 | 0.144970 | 7.677892 | 0.0000 |
| D(________04(-2),2) | 0.535955 | 0.098764 | 5.426623 | 0.0000 |
| D(________04(-3),2) | 0.184662 | 0.048076 | 3.841053 | 0.0001 |
|  |  |  |  |  |
|  |  |  |  |  |
| R-squared | 0.810837 | Mean dependent var | | 8.97E-05 |
| Adjusted R-squared | 0.809411 | S.D. dependent var | | 0.054418 |
| S.E. of regression | 0.023757 | Akaike info criterion | | -4.631972 |
| Sum squared resid | 0.224631 | Schwarz criterion | | -4.592207 |
| Log likelihood | 935.0265 | Hannan-Quinn criter. | | -4.616228 |
| Durbin-Watson stat | 2.005271 |  |  |  |
|  |  |  |  |  |
|  |  |  |  |  |

Attached Table 9. Stationarity Test of China Life's Trading Day Returns Without Constants and Trends

| Null Hypothesis: ________05 has a unit root | | | |  |
| --- | --- | --- | --- | --- |
|  |  |  |  |  |
|  |  |  |  |  |
|  |  |  | t-Statistic | Prob.* |
|  |  |  |  |  |
|  |  |  |  |  |
| Augmented Dickey-Fuller test statistic | | | -20.73065 | 0.0000 |
| Test critical values: | 1% level |  | -2.570066 |  |
|  | 5% level |  | -1.941522 |  |
|  | 10% level |  | -1.616231 |  |
|  |  |  |  |  |
|  |  |  |  |  |
| Dependent Variable: D(________05) | | | |  |
|  |  |  |  |  |
|  |  |  |  |  |
| Variable | Coefficient | Std. Error | t-Statistic | Prob. |
|  |  |  |  |  |
|  |  |  |  |  |
| ________05(-1) | -0.956477 | 0.046138 | -20.73065 | 0.0000 |
|  |  |  |  |  |
|  |  |  |  |  |
| R-squared | 0.486837 | Mean dependent var | | 2.05E-06 |
| Adjusted R-squared | 0.486837 | S.D. dependent var | | 0.036182 |
| S.E. of regression | 0.025919 | Akaike info criterion | | -4.465480 |
| Sum squared resid | 0.304323 | Schwarz criterion | | -4.456409 |
| Log likelihood | 1014.664 | Hannan-Quinn criter. | | -4.461906 |
| Durbin-Watson stat | 1.987795 |  |  |  |
|  |  |  |  |  |
|  |  |  |  |  |

Attached Table 10. The first-order difference test of China Life’s daily returns without constant and trend terms

| Null Hypothesis: D(________05) has a unit root | | | | |
| --- | --- | --- | --- | --- |
|  |  |  |  |  |
|  |  |  |  |  |
|  |  |  | t-Statistic | Prob.* |
|  |  |  |  |  |
|  |  |  |  |  |
| Augmented Dickey-Fuller test statistic | | | -13.08657 | 0.0000 |
| Test critical values: | 1% level |  | -2.571348 |  |
|  | 5% level |  | -1.941699 |  |
|  | 10% level |  | -1.616114 |  |
|  |  |  |  |  |
|  |  |  |  |  |
| Dependent Variable: D(________05,2) | | | |  |
|  |  |  |  |  |
|  |  |  |  |  |
| Variable | Coefficient | Std. Error | t-Statistic | Prob. |
|  |  |  |  |  |
|  |  |  |  |  |
| D(________05(-1)) | -4.309329 | 0.329294 | -13.08657 | 0.0000 |
| D(________05(-1),2) | 2.517721 | 0.302523 | 8.322415 | 0.0000 |
| D(________05(-2),2) | 1.864282 | 0.261400 | 7.131918 | 0.0000 |
| D(________05(-3),2) | 1.243912 | 0.211144 | 5.891302 | 0.0000 |
| D(________05(-4),2) | 0.805576 | 0.154117 | 5.227042 | 0.0000 |
| D(________05(-5),2) | 0.457129 | 0.099701 | 4.585016 | 0.0000 |
| D(________05(-6),2) | 0.171646 | 0.048276 | 3.555540 | 0.0004 |
|  |  |  |  |  |
|  |  |  |  |  |
| R-squared | 0.804371 | Mean dependent var | | -0.000159 |
| Adjusted R-squared | 0.801074 | S.D. dependent var | | 0.061557 |
| S.E. of regression | 0.027455 | Akaike info criterion | | -4.333442 |
| Sum squared resid | 0.268344 | Schwarz criterion | | -4.258344 |
| Log likelihood | 793.5198 | Hannan-Quinn criter. | | -4.303591 |
| Durbin-Watson stat | 2.067374 |  |  |  |
|  |  |  |  |  |
|  |  |  |  |  |

Attached Table 11. Stationarity Test of Ping An’s Daily Returns Without Constants and Trends

| Null Hypothesis: ________06 has a unit root | | | |  |
| --- | --- | --- | --- | --- |
|  |  |  |  |  |
|  |  |  |  |  |
|  |  |  | t-Statistic | Prob.* |
|  |  |  |  |  |
|  |  |  |  |  |
| Augmented Dickey-Fuller test statistic | | | -22.86672 | 0.0000 |
| Test critical values: | 1% level |  | -2.570066 |  |
|  | 5% level |  | -1.941522 |  |
|  | 10% level |  | -1.616231 |  |
|  |  |  |  |  |
|  |  |  |  |  |
| Dependent Variable: D(________06) | | | |  |
|  |  |  |  |  |
|  |  |  |  |  |
| Variable | Coefficient | Std. Error | t-Statistic | Prob. |
|  |  |  |  |  |
|  |  |  |  |  |
| ________06(-1) | -1.037806 | 0.045385 | -22.86672 | 0.0000 |
|  |  |  |  |  |
|  |  |  |  |  |
| R-squared | 0.535807 | Mean dependent var | | -2.85E-06 |
| Adjusted R-squared | 0.535807 | S.D. dependent var | | 0.022706 |
| S.E. of regression | 0.015470 | Akaike info criterion | | -5.497662 |
| Sum squared resid | 0.108409 | Schwarz criterion | | -5.488591 |
| Log likelihood | 1248.969 | Hannan-Quinn criter. | | -5.494088 |
| Durbin-Watson stat | 1.985909 |  |  |  |
|  |  |  |  |  |
|  |  |  |  |  |

Attached table 12. First-order difference test of Ping An’s daily returns without constant and trend terms

| Null Hypothesis: D(________06) has a unit root | | | | |
| --- | --- | --- | --- | --- |
|  |  |  |  |  |
|  |  |  |  |  |
|  |  |  | t-Statistic | Prob.* |
|  |  |  |  |  |
|  |  |  |  |  |
| Augmented Dickey-Fuller test statistic | | | -17.00417 | 0.0000 |
| Test critical values: | 1% level |  | -2.570727 |  |
|  | 5% level |  | -1.941613 |  |
|  | 10% level |  | -1.616171 |  |
|  |  |  |  |  |
|  |  |  |  |  |
| Dependent Variable: D(________06,2) | | | |  |
|  |  |  |  |  |
|  |  |  |  |  |
| Variable | Coefficient | Std. Error | t-Statistic | Prob. |
|  |  |  |  |  |
|  |  |  |  |  |
| D(________06(-1)) | -3.016692 | 0.177409 | -17.00417 | 0.0000 |
| D(________06(-1),2) | 1.161229 | 0.146958 | 7.901755 | 0.0000 |
| D(________06(-2),2) | 0.528239 | 0.099088 | 5.331006 | 0.0000 |
| D(________06(-3),2) | 0.175379 | 0.047466 | 3.694862 | 0.0003 |
|  |  |  |  |  |
|  |  |  |  |  |
| R-squared | 0.814941 | Mean dependent var | | 0.000149 |
| Adjusted R-squared | 0.813546 | S.D. dependent var | | 0.039618 |
| S.E. of regression | 0.017107 | Akaike info criterion | | -5.288725 |
| Sum squared resid | 0.116478 | Schwarz criterion | | -5.248960 |
| Log likelihood | 1067.034 | Hannan-Quinn criter. | | -5.272981 |
| Durbin-Watson stat | 2.005468 |  |  |  |
|  |  |  |  |  |
|  |  |  |  |  |
